# A direct interaction between NQO1 and a chemotherapeutic dimeric naphthoquinone

**DOI:** 10.1186/s12900-016-0052-x

**Published:** 2016-01-28

**Authors:** Lakshmi Swarna Mukhi Pidugu, J.C. Emmanuel Mbimba, Muqeet Ahmad, Edwin Pozharski, Edward A. Sausville, Ashkan Emadi, Eric A. Toth

**Affiliations:** Department of Biochemistry and Molecular Biology, University of Maryland School of Medicine, Baltimore, MD 21201 USA; Marlene and Stewart Greenebaum Cancer Center, University of Maryland School of Medicine, Baltimore, MD 21201 USA; Institute for Bioscience and Biotechnology Research, and Center for Biomolecular Therapeutics, 9600 Gudelsky Drive, Rockville, MD 20850 USA

**Keywords:** NQO1, Dimeric naphthoquinone, Oxidative stress, Anti-cancer agents

## Abstract

**Background:**

Multimeric naphthoquinones are redox-active compounds that exhibit antineoplastic, antiprotozoal, and antiviral activities. Due to their multimodal effect on perturbation of cellular oxidative state, these compounds hold great potential as therapeutic agents against highly proliferative neoplastic cells. In our previous work, we developed a series of novel dimeric naphthoquinones and showed that they were selectively cytotoxic to human acute myeloid leukemia (AML), breast and prostate cancer cell lines. We subsequently identified the oxidoreductase NAD(P)H dehydrogenase, quinone 1 (NQO1) as the major target of dimeric naphthoquinones and proposed a mechanism of action that entailed induction of a futile redox cycling.

**Results:**

Here, for the first time, we describe a direct physical interaction between the bromohydroxy dimeric naphthoquinone E6a and NQO1. Moreover, our studies reveal an extensive binding interface between E6a and the isoalloxazine ring of the flavin adenine dinucleotide (FAD) cofactor of NQO1 in addition to interactions with protein side chains in the active site. We also present biochemical evidence that dimeric naphthoquinones affect the redox state of the FAD cofactor of NQO1. Comparison of the mode of binding of E6a with those of other chemotherapeutics reveals unique characteristics of the interaction that can be leveraged in future drug optimization efforts.

**Conclusion:**

The first structure of a dimeric naphthoquinone-NQO1 complex was reported, which can be used for design and synthesis of more potent next generation dimeric naphthoquinones to target NQO1 with higher affinity and specificity.

**Electronic supplementary material:**

The online version of this article (doi:10.1186/s12900-016-0052-x) contains supplementary material, which is available to authorized users.

## Background

Multimeric naphthoquinones are reduction/oxidation (redox)-active compounds that possess a wide array of therapeutic activities. In particular, these compounds have exhibited well tolerated antibacterial, antifungal, antiviral, and antithrombotic activities [1]. One of the most notable members of this class of compounds is conocurvone, a naturally-occurring trimeric naphthoquinone with a potent anti-HIV activity [2]. Synthetic and natural naphthoquinones have demonstrated significant antineoplastic activity against hematologic and solid malignant cells [3–5]. In an effort to regiospecifically synthesize conocurvone, we previously developed a series of novel dimeric naphthoquinones and showed that they were selectively cytotoxic to human acute myeloid leukemia (AML), breast and prostate cancer cell lines and in particular those cell lines that rely on oxidative phosphorylation [6–8]. To better understand the mechanism of action of these agents, we performed a chemical genetic screen in yeast and identified the yeast oxidoreductase Nde1 as the major target of dimeric naphthoquinones [6, 9]. The human homologue of Nde1 is NAD(P)H quinone oxidoreductase 1 (E.C. 1.6.99.2, hereon referred to as NQO1, also known as DT-diaphorase and NAD(P)H dehydrogenase, quinone 1).

NQO1 is a quinone detoxifying flavoenzyme that catalyzes the two-electron reduction of quinones to hydroquinones. For dimeric naphthoquinones, the resulting hydroquinone is highly unstable and spontaneously gives electrons to oxygen and reverts to the oxidized form of quinone, producing two moles of superoxide per one mole of NAD(P)H [10]. The ultimate outcome is a futile redox cycle in NQO1-overexpressing cells, such as many cancer cells, which can culminate in formation of substantial reactive oxygen species (ROS), oxidative damage to DNA and single- and double-strand DNA breaks. NQO1 exists as a homodimer with two tightly-associated flavin adenine dinucleotide (FAD) cofactors that reside at the deepest point of each active site of two monomers of 274 residues [11]. The two active sites reside at opposite ends of the dimer and incorporate residues from each monomer. The normal biological function of NQO1 is to protect cells from the mutagenic, cytotoxic, and carcinogenic effects of natural and synthetic quinones [12]. The obligate two electron reduction performed by NQO1 averts one-electron reduction of quinones by other flavoproteins such as cytochrome P450, which produces highly reactive radical semiquinone.

The role of NQO1 in cancer varies due to its role in redox biology. NQO1 exhibits tumor suppressor properties by modulating the stability of p53 [13, 14] and participating in suppression of the inflammatory response [15]. Conversely, increased expression of NQO1 can confer a growth advantage in some cancers such as melanoma, pancreatic adenocarcinoma, non-small cell lung cancer, and prostate cancer [16, 17]. The association between the NQO1 C609T polymorphism and increased risk of AML and acute lymphoblastic leukemia (ALL) has also been reported [18, 19]. Exploitation of NQO1 as a target for cancer therapy typically entails two strategies. In some cases, inhibition of NQO1 can suppress cancer cell growth and potentiate chemotherapeutic cytotoxicity [20]. In other cases, NQO1 can be used to activate particular quinone-based chemotherapeutics via its redox activity [21, 22]. For dimeric naphthoquinones, we have proposed that their unique chemical structures undergo NQO1-dependent redox cycling that produces an insurmountable amount of ROS that ultimately lead to mitochondrial dysfunction, DNA damage and cell death [9].

In the present study, we have determined the crystal structure of the novel dimeric naphthoquinone, 3-bromo-3′-hydroxy-2,2′-binaphthalenyl-1,4,1′,4′-tetraone (E6a [23], Additional file 1: Figure S1) bound to NQO1. This structure represents the first evidence of a direct interaction between a dimeric naphthoquinone and NQO1. Moreover, we present biochemical evidence that this interaction affects the redox state of the FAD cofactor. Our structure reveals an extensive binding interface between E6a and the isoalloxazine ring of the FAD cofactor of NQO1 in addition to interactions with protein side chains in the active site. This structure can be used as a starting point to design and synthesize more potent dimeric naphthoquinones tailored to target NQO1 with high affinity and specificity.

## Results and discussion

### Overall quality of crystal structure

Crystals of hNQO1-FAD (holo-hNQO1) belong to space group P2_1_ and diffract to 2.0 Å resolution. The asymmetric unit contains two dimers. The crystal structure shows excellent stereochemistry with 96 % of residues in the most favored region of Ramachandran plot. Each physiological dimer [11, 24–27] is made up of two subunits that are related by a non-crystallographic two fold axis. The overall structure of the dimer is almost identical to that of a previously-determined holo-NQO1 structure (PDB accession code: 1D4A [26]) with a root-mean-square deviation (rmsd) of 0.1 Å. We observe unexplained Fo-Fc density in the active site which does not belong to any of the possible ingredients of protein purification and/or crystallization. When we try to model benzoic acid in this density, it refines to partial occupancy (0.5). The crystal structure of the hNQO1-FAD-E6a complex was determined to a resolution of 2.9 Å with excellent stereochemistry (Table 1) as 93 % of the residues reside in the most favored region of Ramachandran plot. The overall structure shows interpretable electron density for most of the polypeptide chain and five bound E6a molecules. The asymmetric unit of this P2_1_2_1_2_1_ crystal form contains seven physiological dimers. Each physiological dimer is formed by two subunits related by non-crystallographic symmetry. Residues 1–273 (of 274 for the full-length protein) are visible in each monomer. The overall structure of each of the seven dimers is nearly identical to holo-hNQO1 except for a few changes in residues at the active site region interacting with E6a and the isoalloxazine ring of FAD. The Cα superposition of seven dimers onto those of the holo-hNQO1 (above) and to that of 1D4A resulted in rmsd ranging from 0.26 to 0.27 Å. In this crystal form, a considerable amount of surface area is buried by the inter-dimer interactions (>1000 Å^2^ for all but one pair) with neighboring dimers within the asymmetric unit as well as those related by crystallographic symmetry.

**Table 1:** Data collection and refinement statistics

	hNQO1 Apo	hNQO1+E6a
Space Group	P2_1_	P2_1_2_1_2_1_
Unit Cell (Å)	a = 56.93	a = 95.60
	b = 107.16	b = 210.77
	c = 99.76	c = 228.08
	β = 100.68	
Resolution (Å)	2.01	2.9
Unique Reflections	72707	102839
Multiplicity (Last shell)	2.7 (2.5)	11.7 (10.4)
Completeness (Last shell)	96.3 (92.7)	100 (99.9)
R_pim_	0.09	0.116
Refinement		
R_work_	18.0	18.3
R_free_	21.6	22.0
Rms Bond/angle	0.01/1.7	0.01/1.1

**Fig. 1:**
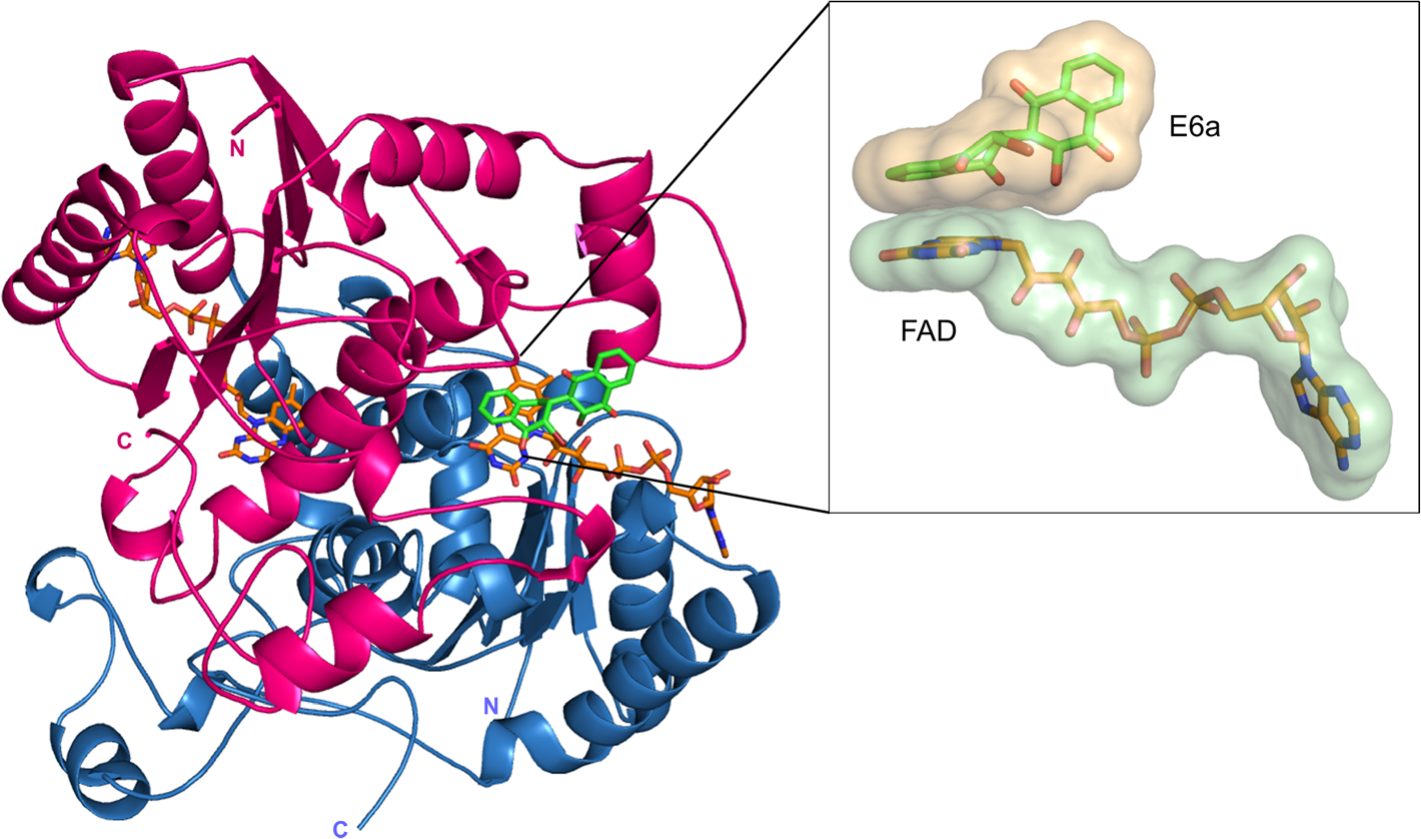
The biological dimer of hNQO1 with two active sites, one at each end of the dimer interface. One monomer is colored magenta while the other monomer is colored blue. Two FAD molecules present at each active site are shown orange and an E6a molecule is shown in green. The inset shows the surface area buried upon FAD-E6a interaction

**Fig. 2:**
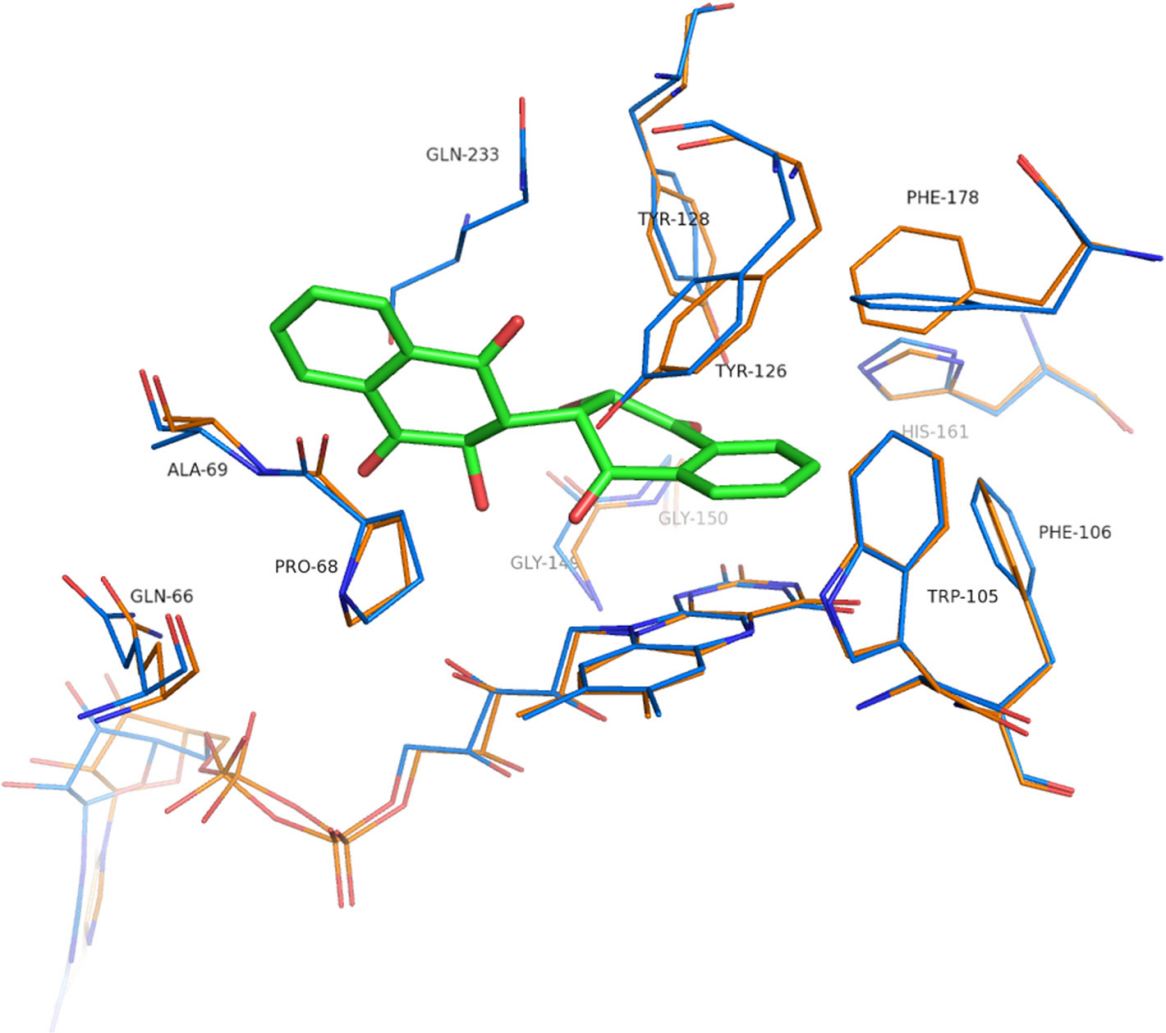
Conformational changes at the active site of holo (orange) and E6a bound (blue) hNQO1

Each subunit in the physiological dimer contains a catalytic domain (1–220) and a C-terminal domain (221–273). Each dimer of hNQO1 has two active sites formed at opposite ends of the dimer interface (Fig. 1). Each catalytic domain has a bound FAD molecule with its adenine ring interacting mainly with the catalytic domain and isoalloxazine ring present at the dimer interface. The flavin of the FAD forms the floor of the catalytic site while the residues Trp105, Phe106, Gly149, Gly150, Tyr155, His161 from one subunit contribute to hydrophobic walls and Tyr126′, Tyr128′ and Phe178′ from the other subunit make up the roof. The noticeable differences at the active site of holo-hNQO1, when compared to that of 1D4A, include differences in the side chain orientations of Phe106, Tyr128′, Phe178′ and Phe232′ (Additional file 2: Figure S2). There are no significant changes in the overall structure upon E6a binding with the exception that the loop containing active site residues (main chain of residues 127–130) moves about 1.2–1.5 Å (measured at Cα of Tyr128′) towards the active site contributing to the slight shift in the position of Tyr128′. Residues Phe232 and Gln233 from a loop (230–236) face away from the dimer and interact with the active site of neighboring dimer (Additional file 3: Figure S3). As this loop competes for space with that of the active site loop containing Tyr128′, only the side chain of either Tyr128′ or Gln233″ (i.e. not both) are ordered in each active site. Tyr128′ from subunit K and corresponding Gln233″ are well-ordered in the active site containing E6a. Hence this active site was used for the analysis and making figures. There are no significant changes in the overall structures of these complexes when compared to the holo form except for the active site residues Tyr126′ Tyr128′ and Phe178′ (Fig. 2). FAD binding is identical to that of the holo form. This is consistent with previously reported crystal structures, which show that the substrates NADH, chemotherapeutic quinones and coumarin-based inhibitors bind at this active site with minor differences in active site architecture [11, 28–30].

### E6a interactions with the NQO1 active site

**Fig. 3:**
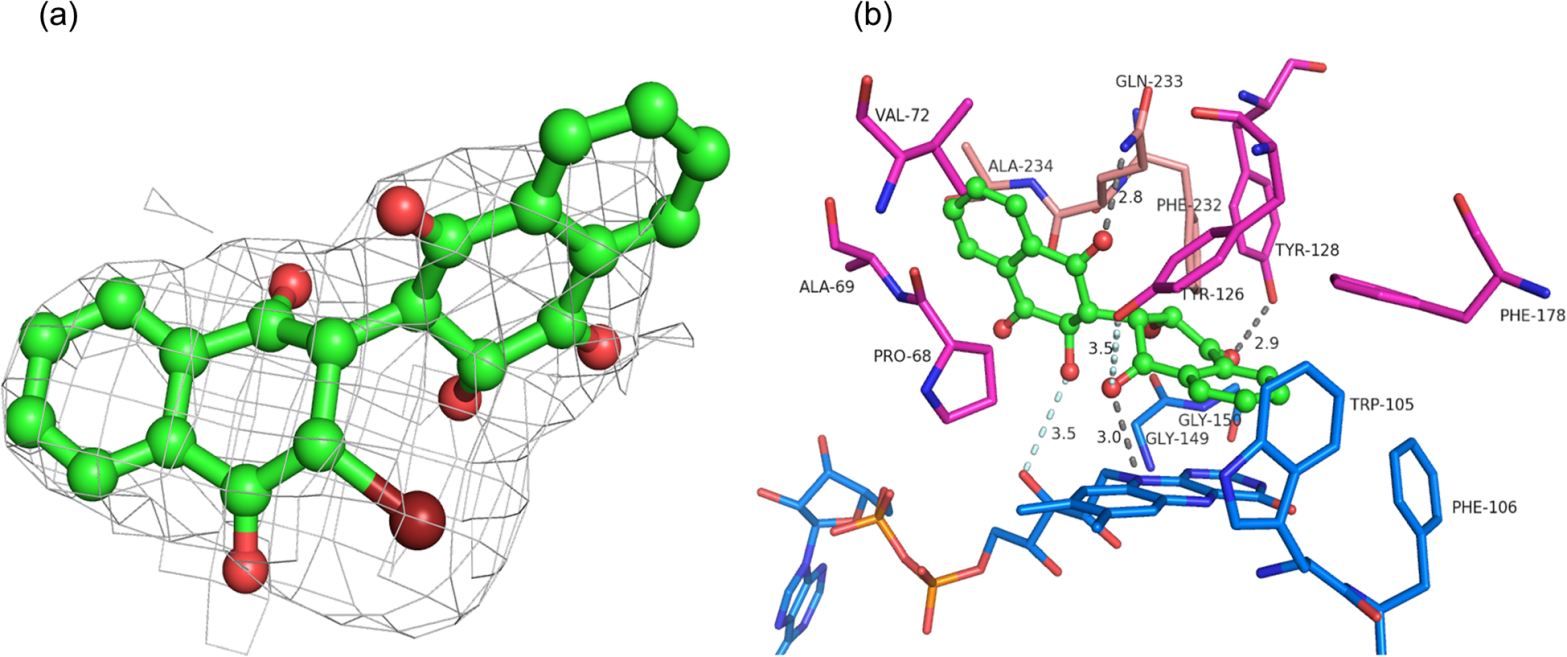
**a** 2Fo-Fc electron density for E6a contoured at 1σ. **b** Interactions of E6a with the active site residues of hNQO1. The residues from one subunit are represented in blue while the second subunit is shown in magenta. E233 from neighboring dimer is shown in light pink. The hydrogen bonds are shown in gray and weak electrostatic interactions in pale blue

E6a binds in the active site at the same site as the nicotinamide ring of NADH as observed in a previous study [29]. Five out of 14 active sites show clear density for most atoms of the dimeric naphthoquinone (Fig. 3a). The E6a was modelled into the electron density at the active site by positioning its Br atom in the Fo-Fc map contoured at 5σ. Two more active sites show clear density for only one ring, i.e., the brominated naphthoquinone ring. Partial density is visible for the hydroxyl-containing naphthoquinone ring. Approximately 116.8 Å^2^ of surface area of FAD is buried upon E6a binding. This is almost two fifths of the surface area buried by protein atoms in the interaction (299.0 Å^2^). This indicates that interactions with FAD contribute substantial favorable energy to the interaction with E6a. E6a binds with its halogenated naphthoquinone ring (Ring A) stacking against the isoalloxazine ring of FAD with the remainder of the compound interacting with the residues from both subunits of the dimer. The distance between the isoalloxazine ring and the brominated ring of E6a is approximately 3.7 Å. In this position the quinone core of E6a interacts mostly with A and B rings of the flavin. Most of the interactions of brominated ring of E6a with FAD are hydrophobic except for a hydrogen bond between O19 of E6a and hydrogen attached to N10 of the central ring of the isoalloxazine moiety and a weak electrostatic interaction of 3.5 Å between O41 of E6a and O3′ of FAD (Fig. 3b). The brominated ring of E6a sits in a hydrophobic pocket lined by Trp105, Phe106, Phe178′, Tyr126′ and Tyr128′. Of these residues, Tyr128′ participates in a hydrogen bond with O20 of E6a while Tyr126′engages in a weak electrostatic interaction (3.5 Å) with O19 of E6a (Fig. 3b). The bromine is held by van der Waals and weak electrostatic interactions ranging from 3.5 to 3.8 Å with the main chain of Gly149 and Gly150. Interestingly, the loop 230–236 from a neighboring dimer, specifically the main chain atoms of Phe232″ and Glu233″, forms the majority of interactions with the other naphthoquinone ring, which contains a hydroxyl group in place of the bromine. Van der Waals interactions with Gln66′, Ala67′, Pro68′ and Val 72′ also help hold this ring in position. To verify this positioning of the second naphthoquinone ring is not an artifact of crystal packing, we co-crystallized hNQO1 with E6a. These crystals show poor diffraction to 3.5 Å and belong to the space group P4_1_2_1_2. This crystal form has a different packing where we do not see the loop 230–236 of the neighboring molecules interacting with the active site (data not shown). This crystal form also shows a similar binding mode for E6a, confirming that binding mode and orientations of the two naphthoquinone rings are unique and independent of the crystal form. Movement of the main chain of loop 230–236 and side chain of Phe232 is to accommodate the substrates and is mentioned in previously reported structures of NQO1 complexed with dicoumarol (2F1O), duroquinone (1QRD) and ES936 (1KBQ) [28, 29, 31]. It was also shown that a specific conformation of Tyr128′ and Phe232′ is important for NQO1 interaction with p53 and the movement in these residues upon the binding to dicoumarol changes the surface properties of hNQO1, rendering it incapable of binding to its client proteins like p53 and p73β [11].

### Increase in FAD fluorescence upon E6a binding

**Fig. 4:**
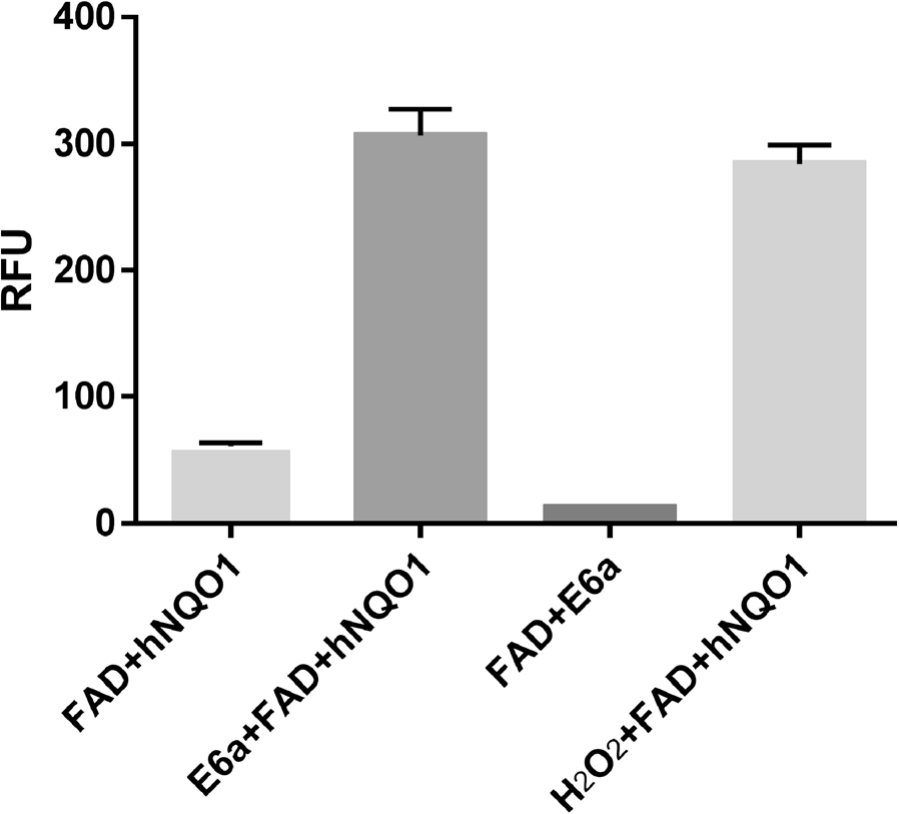
Increase in hNQO1+FAD fluorescence upon E6a binding. The relative fluorescence units (RFU) of each sample were measured in triplicate and the standard deviations are represented as error bars. Unpaired *t*-test analysis of hNQO1+FAD+E6a versus hNQO1+FAD+H_2_O_2_ resulted in a p value of 0.212 while all other combinations gave *p* values less than 0.001

FAD, the co-factor of hNQO1, is naturally fluorescent. Fluorescence of FAD bound to several proteins like flavodoxin, lactate oxidase, etc. in its oxidized state exhibits much higher fluorescence intensity than that of its reduced state [32, 33]. In the case of cholesterol oxidase, on and off states of FAD fluorescence were observed as the redox state of flavin toggles between oxidized (FAD) and reduced states (FADH_2_) [34]. Interestingly, the fluorescence of FAD bound to hNQO1 [61.1 +/− 1. 7 relative fluorescence Units (RFU) (*n* = 3)] showed a 5-fold increase [306.3 +/− 12.1 RFU (*n* = 3)], upon E6a binding (Fig. 4). Fluorescence of E6a alone at an excitation of 425 nm and emission of 525 nm was negligible. This increase in FAD fluorescence could be attributed to the oxidation of hNQO1-FADH_2_ to hNQO1-FAD upon E6a binding, which in turn results in conversion of E6a to its hydronaphthoquinone counterpart. The reduced form of E6a, i.e. hydronaphthoquinone, can quickly cycle back to oxidized form of E6a by generating ROS including superoxide and peroxide. As a positive control, we observed an increase in fluorescence of FAD bound to hNQO1 in presence of hydrogen peroxide (H_2_O_2_) to 284.2 +/− 8.6 RFU (*n* = 3). The fluorescence of FAD and E6a in the absence of protein is very low [12.52 +/− 0.1 RFU (*n* = 3)]. These results confirm that oxidation of FAD by E6a occurs only in the context of the hNQO1-FAD complex. Our previous results showed that in yeast, dimeric naphthoquinones generate ROS in a concentration dependent manner [9]. The FAD fluorescence results are consistent with our previous prediction that dimeric naphthoquinones engage hNQO1 in futile redox cycling and generation of ROS.

### Comparison of E6a binding to that of other quinone-based chemotherapeutic agents

**Fig. 5:**
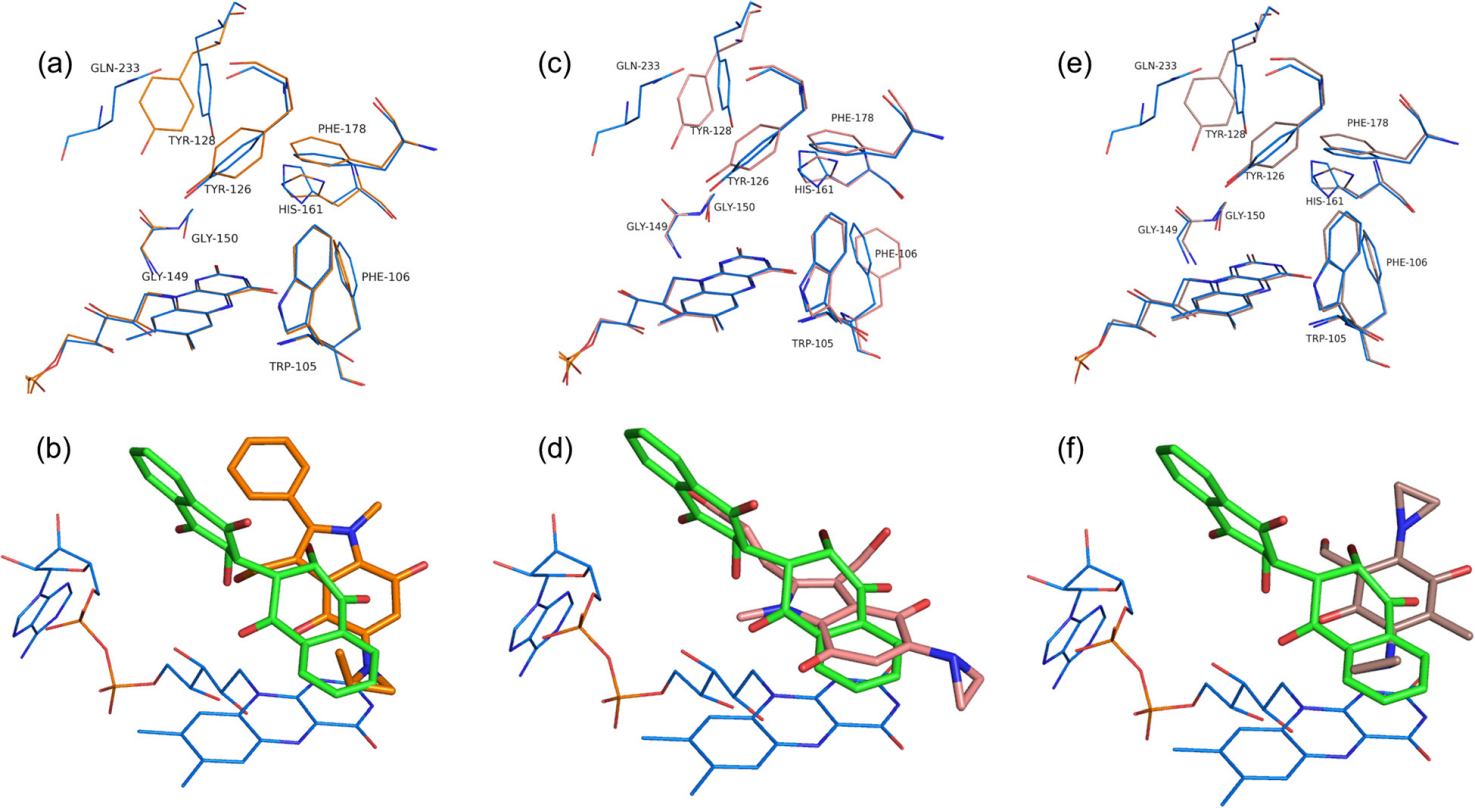
Comparison of the mode of binding of E6a to that of other chemotherapeutic quinones. Residues from the hNQO1-E6a complex are shown in blue and E6a is shown in green. Residues from the quinone complex structures are shown in orange (ARH019), pink (E09), and brown (RH1). **a**, **c** and **e** depict the conformational changes in the active site residues while (**b**), (**d**) and (**f**) show variations of binding modes of E6a and quinone-based chemotherapeutic agents ARH019 (*orange*), E09 (*pink*) and RH1 (*brown*), respectively

E6a binds in the same active site pocket as that of the chemotherapeutic quinones but in a different orientation (Fig. 5). The brominated naphthoquinone ring occupies a similar position and orientation as that of the aromatic core of the chemotherapeutic quinones RH1 (PDB Code: 1H66, Additional file 4: Figure S4) and ARH019 (PDB Code: 1H69, Additional file 4: Figure S4) (Fig. 5b, f), but shifted by 1.4 and 1.9 Å, with respect to RH1 and ARH019, respectively, towards the central ring of the flavin and away from His161 and Phe178′. In this position, the benzene ring of the brominated naphthoquinone ring has interactions with Trp105 and Tyr126′ similar to those of the aziridinyl group of RH1 and ARH019. In contrast to RH1 and ARH019, the quinone oxygen O19 of E6a is hydrogen bonded to Tyr126′ but not to Tyr128′. Instead, the quinone oxygen O20 of E6a is hydrogen bonded to Tyr128′, the side chain of which occupies a different position when compared to the structures of other quinone-based chemotherapeutics (Fig. 5a, c, e). In this position, oxygen O20 of E6a is 4.5 Å from His161, a residue that forms a hydrogen bonding interaction with RH1, ARH019, and E09 (discussed below). The other naphthoquinone ring of E6a is positioned in a different pocket lined by main chain of Gln66-Pro68 when compared to the 2-phenyl group of ARH019 stacking with Gly149 and Gly150. The bromine of E6a interacts with Gly149 and Gly150. In contrast to the other quinones, the quinone ring of E09 (PDB Code: 1GG5, Additional file 4: Figure S4) aligns with the naphthyl ring of E6a and the indole ring of E09 aligns with the brominated quinone ring of E6a (Fig. 5d). This shift relative to E6a appears to be facilitated in part by favorable van der Waals interactions created with the side chains of residues Phe178, Phe106′, and Trp105′ and the aziridinyl ring of E09. In addition, hydrogen bonds from His161′ and Tyr126 to the quinone oxygen of E09 appear to stabilize this orientation. In the E6a structure, only the van der Waals contacts between Phe178 and the naphthyl ring of E6a are maintained relative to the E09 structure. The main differences in the active site residues include the orientation differences in the side chains of His161, Tyr126′, Tyr128′ and Phe178′. Tyr128′ shows a large swing in its side chain away from the active site to accommodate the second naphthoquinone ring. The loop 230–236 from a neighboring dimer interacting with E6a bound in the active site could also contribute to this position of Tyr128′. These structures of chemotherapeutic quinones clearly show that these compounds, even the ones with highly similar pharmacophores, can “float” along the isoalloxazine ring of FAD and bind to the enzyme in different orientations as dictated by interactions with side chains in the active site [30]. The smaller size of these compounds when compared to the active site pocket gives them flexibility to migrate to positions best suited for their substitutions at various positions. This property might make optimization and/or rational design quite difficult because chemical modification of the compounds could change their binding orientation, thereby thwarting the intention of the design. On the other hand, the dimeric naphthoquinones are bulky, occupy most of the active site, and appear to have little room for changing orientations. This opens up the possibility for a design in which the halogenated naphthoquinone ring is anchored at the FAD and the other naphthoquinone ring can be optimized to improve efficacy.

### Comparison of E6a binding to coumarin-based inhibitors of NQO1

**Fig. 6:**
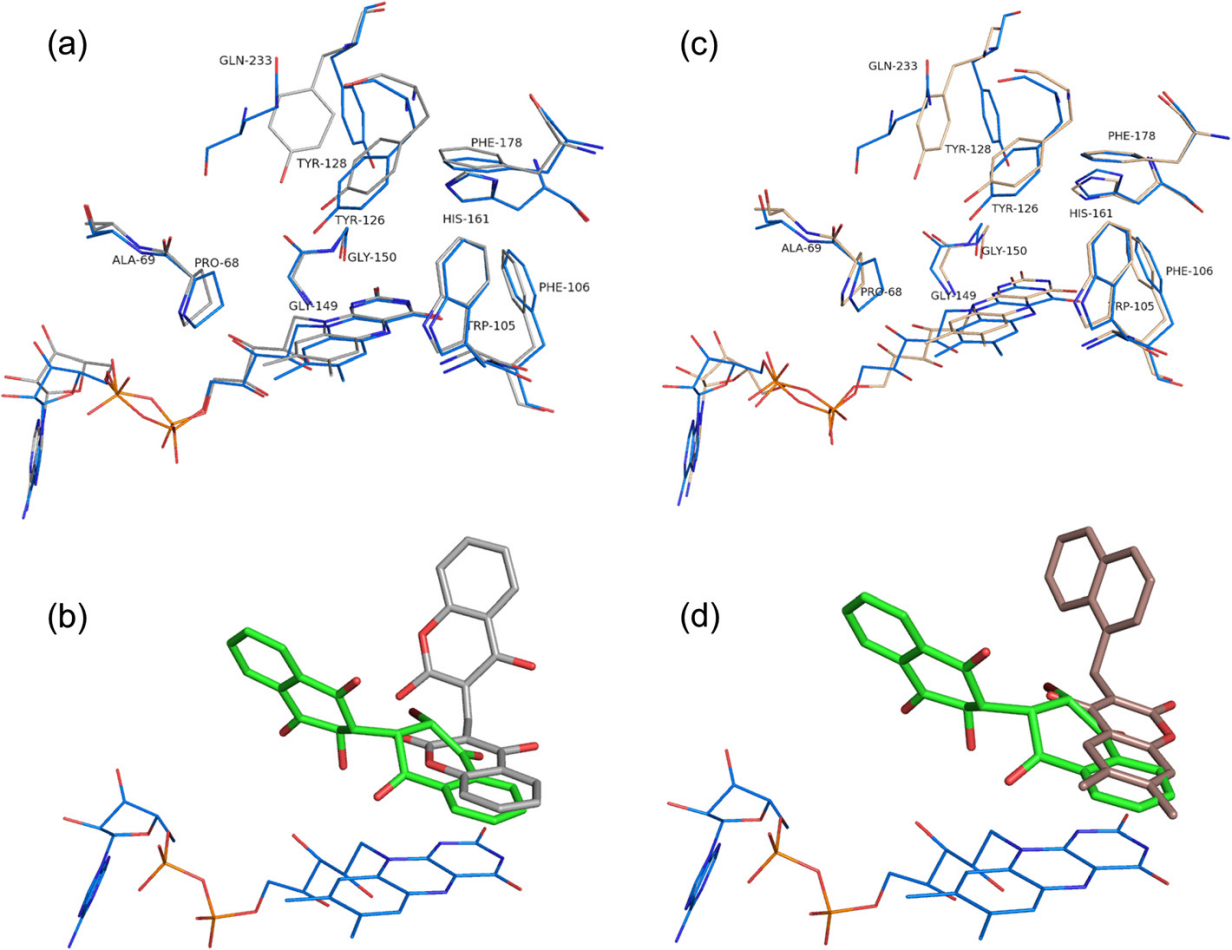
Comparison of E6a and coumarin-based NQO1 inhibitors binding to the active site of hNQO1. Residues from the hNQO1-E6a complex are shown in blue and E6a is shown in green. Residues from the coumarin complexes are shown in *gray* (dicoumarol) and brown (AS1). **a** and **c** show the structural differences in the active site residues of hNQO1-dicoumarol and hNQO1-AS1 with reference to the hNQO1-E6a complex. **b** and **d** show the differences in binding orientations of dicoumarol (*gray*) and AS1 (*brown*) compared to that of E6a

In contrast to the other therapeutic quinones, the position and orientation of bound E6a differs markedly when compared to those of coumarin-based inhibitors of NQO1 including dicoumarol (PDB Code: 2F1O) and AS1 (PDB Code: 3JSX, Additional file 4: Figure S4) (Fig. 6). The coumarin ring of dicoumarol that stacks against the isoallozaxine ring of FAD is stabilized by the hydrogen bonds between O5 and Tyr128′ and O17 and NE2 of His161. The other coumarin ring is stacked against Tryr128′ and is held in position by hydrogen bonds between O38-His161 and O32-Gly149 main chain. The crystal structure of hNQO1-AS1 complex shows that the coumarin ring of AS1 that stacks against the flavin ring binds in the same position but in a different orientation to that of dicoumarol (Fig. 6b), with its hydroxyl group at position 4 interacting with Tyr128′ while O2 and O7 are hydrogen bonded to His161 (Fig. 6a). This is clearly due to the methyl substitutions at positions C1 and C6 of this coumarin ring. The naphthyl group of AS1 occupies similar position to that of the second coumarin ring of dicoumarol but in a perpendicular direction interacting mainly with Gly149, Gly150, Met154, Phe232′ and Tyr128′ (Fig. 6d). In this crystal structure, the naphthyl ring interacts with Phe232″ and Glu233″ of the neighboring dimers due to crystal packing, with the distances ranging from 3.5 to 5.8 Å in eight monomers present in the asymmetric unit. The overall binding pocket but not orientation of the brominated naphthoquinone ring of E6a matches that of the coumarin ring of dicoumarol and AS1 that stacks against the flavin, mirroring the van der Waals interactions with Tyr126′, Phe178 and Phe106. However the hydrogen bonding pattern differs in the case of E6a. The striking difference in E6a binding compared to that of the coumarin-based derivatives is the positioning of the second naphthoquinone ring in a pocket lined by the main chains of Gln66-Pro68, phosphates of FAD. In the second ring of dicoumarol and AS1, both occupy a different pocket which resides 8.0 Å away from the pocket occupied by the corresponding ring on E6a. Other differences in the active site include the conformation of side chains of His161, Phe106, His 194, Tyr128′ and Phe232′ (Fig. 6a, c). The mechanism of dicoumarol inhibition involved increasing superoxide levels via inhibition of NQO1 [35–37]. However, other coumarin-based inhibitors show minimal or no superoxide generation though they show efficient inhibition of hNQO1. Dimeric naphthoquinones on the other hand showed a concentration dependent ROS generation in yeast [9]. Our earlier studies on MDA-453 and PC-3 cancer cell lines suggested that generation of reactive oxygen species leading to oxidative stress and mitochondrial dysfunction are the anti-cancer mechanisms of this class of compounds [6, 7].

## Conclusions

The crystal structure of hNQO1 complexed with FAD and E6a presented here is the first evidence of direct interaction of the dimeric naphthoquinones with NQO1 at the active site. This is a valuable starting point for better understanding of the mode of binding of dimeric naphthoquinones to NQO1. Such data are required in order to establish structure activity relationships that support further structure-based optimization to improve the anti-neoplastic efficacy of this novel class of chemotherapeutics. High resolution crystal structures of hNQO1 with more dimeric naphthoquinones would help to understand the complete mechanism of activation of these agents by hNQO1.

## Methods

### Expression and purification

DNA for hNQO1 was codon optimized for expression in *Escherichia coli* and subcloned into a modified pET19b vector containing N-terminal 10XHis tag and PreScission protease cleavage site preceding the insert. The expression plasmid was transformed into BL21 (DE3) cells. Cells were grown at 37 °C to an OD600 of 0.6 and induced with 0.3 mM Isopropyl β-D-1-thiogalactopyranoside (IPTG) at 18 °C overnight. The cells were harvested at 4000 rpm for 20 min and resuspended in lysis buffer containing, 50 mM Tris pH 8.0, 500 mM NaCl and 2 mM beta-mercaptoethanol. The cells were lysed by sonication and soluble proteins were separated by centrifugation at 15000 rpm for 45 min. The clarified lysate was first purified using Ni-affinity chromatography. The histidine tag was removed from the purified hNQO1 by preScission protease cleavage, combined with a dialysis against 50 mM Tris pH 8.0, 150 mM NaCl and 1 mM DTT. Cleaved hNQO1 was further subjected to a final purification step using size exclusion chromatography.

### Crystallization and structure determination

hNQO1 stored at a concentration of approximately 18–20 mg/ml in 50 mM Tris pH 8.0, 50 mM NaCl and 5 μM FAD was used for crystallization screening. Initial screening with JCSG+, Classics suite I and II from Qiagen resulted in initial hits in more than 20 conditions. Native data up to 2.0 Å resolution were collected using P2_1_ crystals obtained in 20 % (w/v) PEG3350 and 0.2 M Ammonium Citrate. Another crystal form in the space group P2_1_2_1_2_1_ were obtained using 20 % PEG3350 and 0.2 M Potassium Sodium Tartarate. The complex between NQO1 and E6a was obtained by soaking the P2_1_2_1_2_1_ native crystals in mother liquor containing 1 mM E6a. X-ray diffraction data were collected in house and at beam line 5.0.3 of the Advanced Light source at Lawrence Berkeley National Laboratory for the holo and E6a-bound hNQO1 crystals respectively. The data were reduced using iMosflm [38] and Aimless [39] from the CCP4 program suite. Initial phases for both holo and E6a-bound hNQO1 were determined by molecular replacement using the program Phaser [40–43] using the coordinates of an hNQO1 monomer from a previously reported holo-structure (PDB accession code: 1D4A) [26]. The initial molecular replacement solution for the hNQO1-E6a complex contained only 8 of the 14 monomers in the asymmetric unit. Using these 8 monomers as a fixed solution, the remaining monomers were placed iteratively using a combination of Phaser [40–43] and Molrep [44]. The structure was refined using Refmac5 [45–49] from CCP4 program suite. Iterative cycles of model building using COOT [50–53] and refinement by refmac5 and TLS- and NCS- restrained [54] refinement using buster [55] yielded final structures with R_work_/R_free_ of 18.0/21.6 for native and 18.3/22.0 for the E6a complex. Final Structures were deposited in PDB (Accession Codes: holo–hNQO1: 5EA2, hNQO1-E6a: 5EAI).

### Fluorescence measurements

All fluorescence measurements were done using SpectraMax M5 plate reader at room temperature. An absorbance scan for 100 μM FAD in 50 mM Tris pH 8.0 and 50 mM NaCl resulted in a peak at 440 nm. The excitation wavelength was chosen to be 425 nm for FAD to avoid overlap of excitation and emission peaks. An emission scan with an excitation wavelength at 425 nm resulted in a peak at 525 nm. These wavelengths were confirmed by performing similar scans with FAD bound to hNQO1 in the same buffer and thus were used for all of the fluorescence experiments. Approximately 100 μl of each of the following samples in 50 mM Tris pH 8.0, 50 mM NaCl and 5 μM FAD were added in the wells of a costar Black/clear bottom 96 well plate. Fluorescence of hNQO1 alone in the above buffer was initially recorded. Next, NQO1 supplemented with 1 mM E6a in the above buffer was analyzed. A mixture of 5 μM FAD and 1 mM E6a mixture in the above buffer was used as a negative control. Approximately 100 μM hydrogen peroxide was added to the NQO1 sample and used as a positive control for the oxidized FAD. These data were analyzed using softMax Pro software.

### Availability of supporting data

The atomic coordinates and structure factor amplitudes are available in the Protein Data Bank repository (PDB), Accession Codes 5ea2 (holo-NQO1) and 5eai (NQO1-E6a complex).

## Additional files

Additional file 1: Figure S1. The chemical structure of E6a (3-bromo-3′-hydroxy-2,2′-binaphthalenyl-1,4,1′,4′-tetraone). (PNG 40 kb) Additional file 2: Figure S2. Superposition of active site residues in holo-hNQO1 structures from the current study (Cyan) and previously reported structure 1D4A (gray). (PNG 1167 kb) Additional file 3: Figure S3. Two dimers of E6a bound hNQO1 structure showing the loop 230–236 interacting with the active site of neighboring dimer. The FAD molecules are shown in stick representation in each active site. The E6a molecule is shown in ball-and-stick representation. (PNG 2799 kb) Additional file 4: Figure S4. The chemical structures of other known inhibitors of NQO1. (PNG 79 kb)

## Abbreviations

ALL: acute lymphoblastic leukemia
AML: acute myeloid leukemia
ARH019: 3-hydroxymethyl-5-(2-methylaziridin-1-yl)-1-methyl-2-phenylindole-4,7-dione
AS1: 4-Hydroxy-6,7-dimethyl-3-(1-naphthylmethyl)-2H-chromen-2-one
E09: 3-hydroxymethyl-5-aziridinyl-1-methyl-2-(H-indole-4,7-indione)-propenol
E6a: 3-bromo-3′-hydroxy-2,2′-binaphthalenyl-1,4,1′,4′-tetraone
FAD: flavin adenine dinucleotide
NAD: nicotinamide adenine dinucleotide
RFU: relative fluorescence units
RH1: 2,5-diaziridinyl-3-hydroxyl-6-methyl-1,4-benzoquinone
ROS: reactive oxygen species
