# Supplementary figures and images for: A direct interaction between NQO1 and a chemotherapeutic dimeric naphthoquinone
Source: BMC Struct Biol. 2016 Jan 28;16:1. doi: 10.1186/s12900-016-0052-x (PMC4730606; doi:10.1186/s12900-016-0052-x)

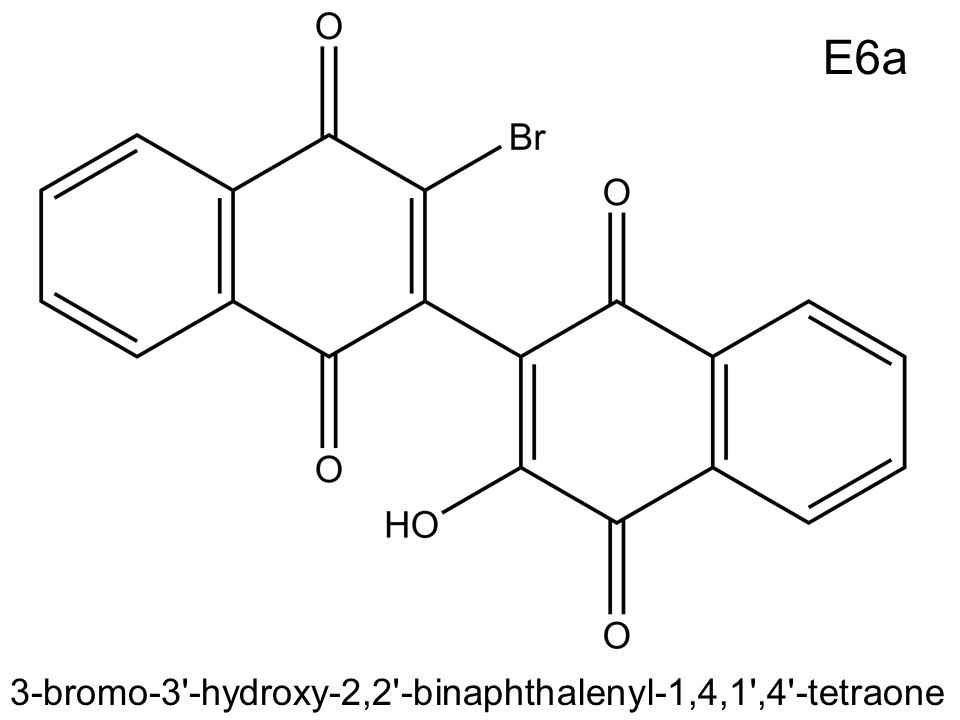

Supplement: Additional file 1: Figure S1. — The chemical structure of E6a (3-bromo-3′-hydroxy-2,2′-binaphthalenyl-1,4,1′,4′-tetraone). (PNG 40 kb) [file 12900_2016_52_MOESM1_ESM.png]

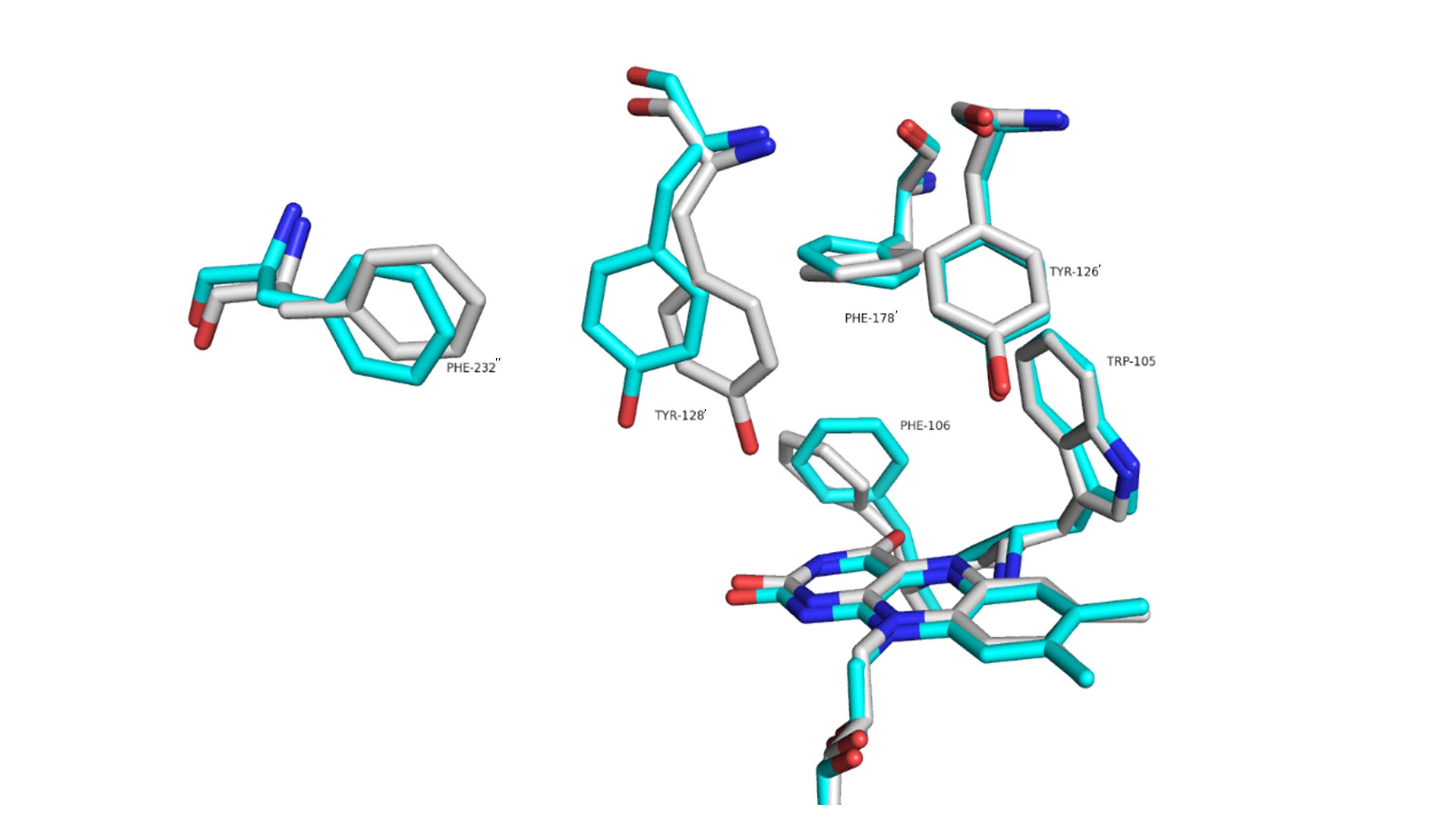

Supplement: Additional file 2: Figure S2. — Superposition of active site residues in holo-hNQO1 structures from the current study (Cyan) and previously reported structure 1D4A (gray). (PNG 1167 kb) [file 12900_2016_52_MOESM2_ESM.png]

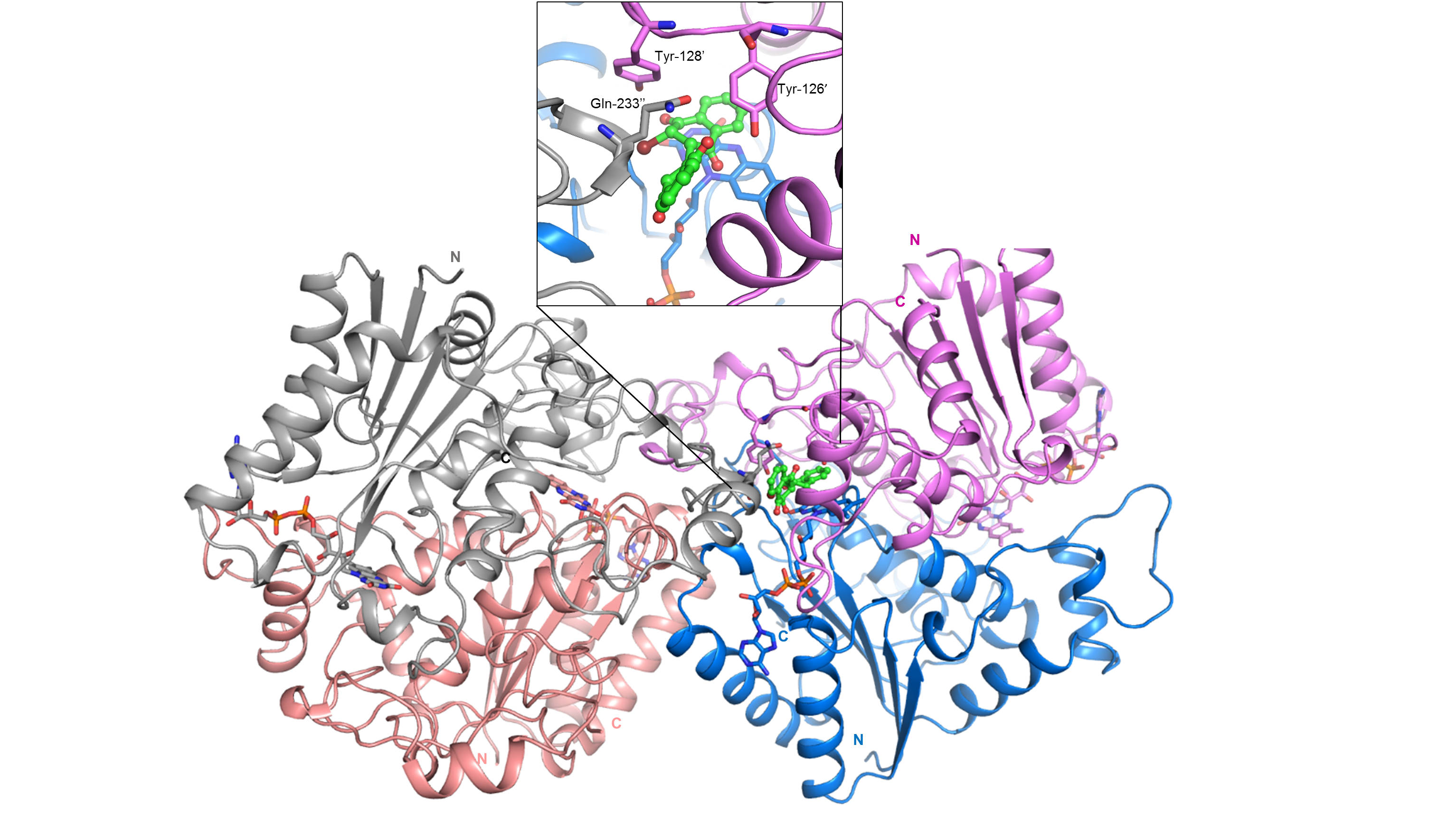

Supplement: Additional file 3: Figure S3. — Two dimers of E6a bound hNQO1 structure showing the loop 230–236 interacting with the active site of neighboring dimer. The FAD molecules are shown in stick representation in each active site. The E6a molecule is shown in ball-and-stick representation. (PNG 2799 kb) [file 12900_2016_52_MOESM3_ESM.png]

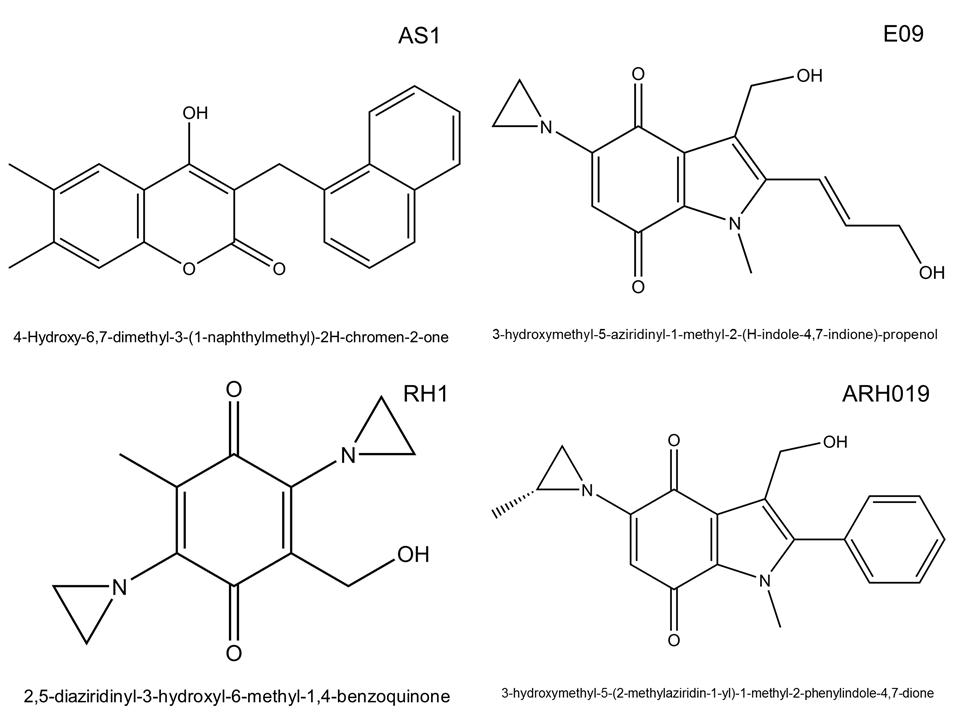

Supplement: Additional file 4: Figure S4. — The chemical structures of other known inhibitors of NQO1. (PNG 79 kb) [file 12900_2016_52_MOESM4_ESM.png]
